# Measurement of scoliosis Cobb angle by end vertebra tilt angle method

**DOI:** 10.1186/s13018-018-0928-5

**Published:** 2018-09-04

**Authors:** Jing Wang, Jin Zhang, Rui Xu, Tie Ge Chen, Kai Sheng Zhou, Hai Hong Zhang

**Affiliations:** 1 Department Of Orthopedics, Orthopedics Key Laboratory of Gansu Province, Lanzhou University Second Hospital, Cuiyingmen, Lanzhou, 730030 Gansu province China; 2 Radiology Department, Lanzhou University Second Hospital, Cuiyingmen, Lanzhou, 730030 Gansu province China

**Keywords:** End vertebra, Tilt angle, Scoliosis, Cobb angle

## Abstract

**Background:**

Scoliosis is a common deformity, and its severity is usually assessed by measuring the Cobb angle on the spinal X-ray film. The measurement of the Cobb angle is an important basis for selecting therapeutic methods and evaluating therapeutic effects. To measure and calculate the scoliosis Cobb angle by end vertebra tilt angle method (tilt angle method) and assess its accuracy and usability.

**Methods:**

It is deduced that the Cobb angle is the sum of upper and lower end vertebra tilt angles through the law of plane geometry. The project included 32 patients with scoliosis who have received treatment in our hospital from June 2011 to July 2016, whose Cobb angles were measured at various segments (total 50). The measuring results of the tilt angle method and the classical method were compared, and the time spent for the measurement of the two groups was respectively recorded with an electronic stopwatch for comparison. The interference of line marking in imaging data pixel in the two groups was compared using Beyond Compare software.

**Results:**

The measuring results through PACS (picture archiving and communication systems) were regarded as the reference standard. There was no statistical difference for measuring the Cobb angle between the PACS method, end vertebra tilt angle method, and classical method. The end vertebra tilt angle method takes less measuring time than the classical method. The measuring error between the classical method and the tilt angle method showed no statistical significance for the difference.

**Conclusion:**

The scoliosis Cobb angle can be measured accurately and rapidly using the principle of the Cobb angle being equal to the sum of tilt angles of the upper and lower end vertebra, where in the film data of imaging will not be easily contaminated. Under special conditions, the average measuring error is ± 3°.

## Background

**Fig. 1:**
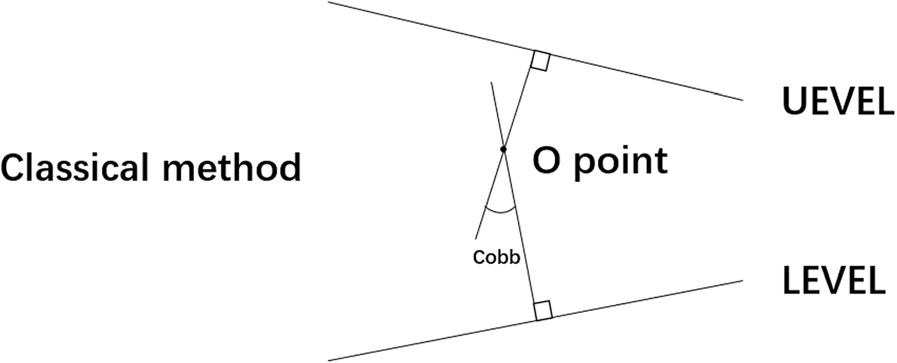
Measurement of Cobb angle by classical method

The scoliosis Cobb angle is an important index of disease assessment. The classical method is used to determine the upper/lower end vertebras (UEV/LEV) on the whole spine anteroposterior X-ray film; then, draw a vertical line respectively at the upper/lower end vertebra endplate lines (UEVEL/LEVEL), and the included angle of the two vertical lines is the Cobb angle [1] (Fig. 1). Manually drawing a line on the image film for measurement is needed in this method and hence is slightly cumbersome, and the line markings can easily contaminate the image data. This study deduced the geometry law of the classical measurement method to calculate the Cobb angle by the end vertebra tilt angle measurement method (tilt angle method) and assess its accuracy, quickness, and contaminated interference in the image data.

## Methods

### General data

This group of patients included 10 males and 22 females, aged 11~24 with an average age of 16. Among which, 22 patients suffered from idiopathic scoliosis, 8 patients suffered from congenital scoliosis, and 2 patients suffered from neuromuscular scoliosis. There were 50 scoliosis segments totally including 24 main thoracic scoliosis segments, 18 thoracolumbar scoliosis segments, and 8 lumbar scoliosis segments.

### Measurement of Cobb angle by PACS

Anteroposterior X-ray of the whole spine was performed on patients to determine the UEV and LEV of scoliosis segments; then, UEVEL and LEVEL were marked on the film using the PACS (picture archiving and communication systems) built-in measuring procedure, and the Cobb angle was automatically calculated. The measurement was made by the same physician. To reduce the self-measuring deviation of the observer, the angle data of each patient was measured three times every 2 weeks and then the average value was taken as the final result.

### End vertebra tilt angle measurement method

**Fig. 2:**
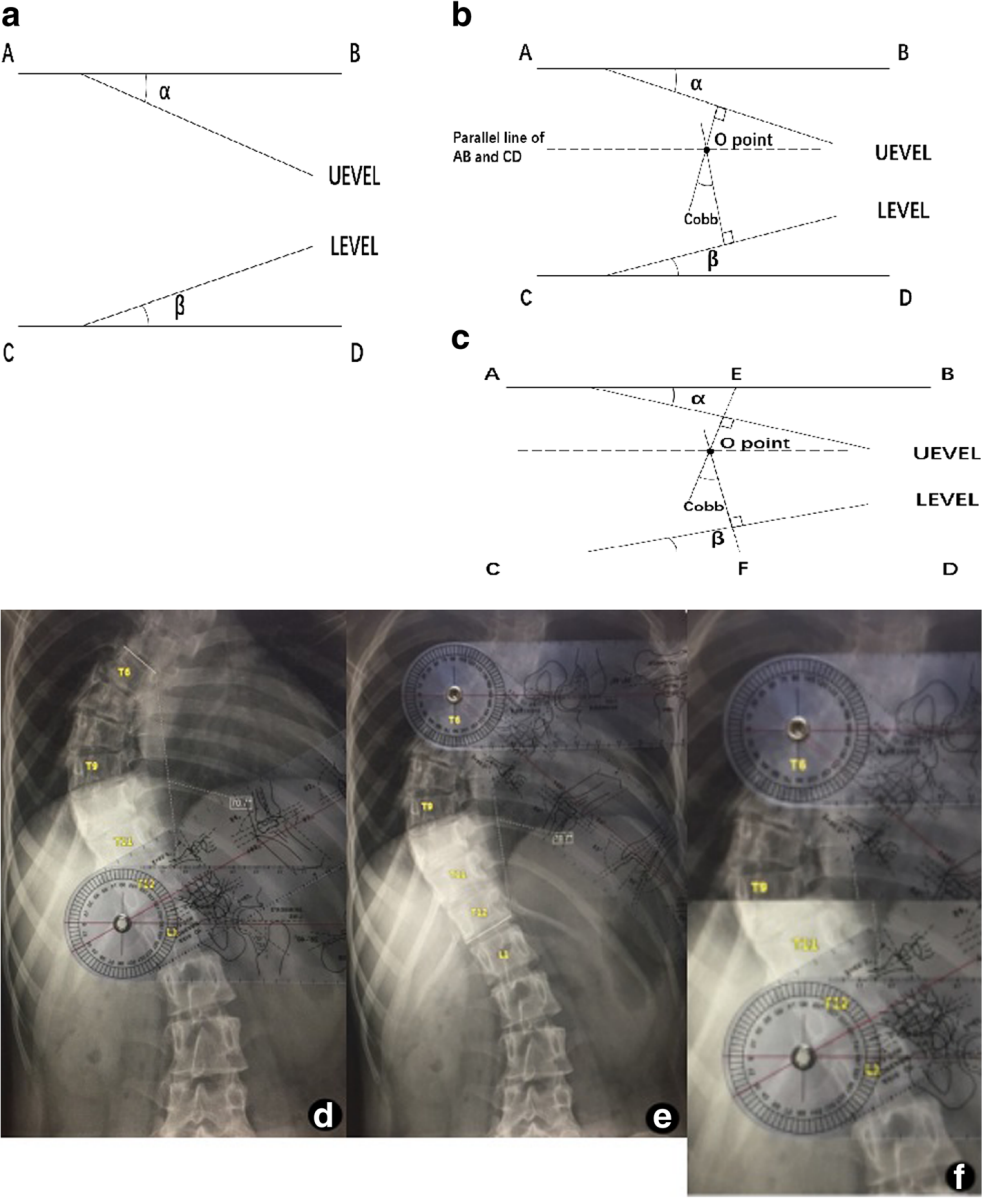
**a**, **b**, **c** Measuring process of the Cobb angle by the end vertebra tilt angle method. **d** Measurement of the upper end vertebra tilt angle. **e** Measurement of the lower end vertebra tilt angle. **f** The upper end vertebra tilt angle *α* = 41°, the lower end vertebra tilt angle *β* = 30°, and the Cobb angle = *α* + *β* = 71°

The classical method for measuring the Cobb angle is to draw a vertical line respectively at UEVEL and LEVEL, and the included angle between the two vertical lines is the Cobb angle. The following auxiliary lines are drawn: the horizontal lines AB and CD. The angle of AB with UEVEL is *α*, and the angle of CD with LEVEL is *β*, which are respectively the upper/lower end vertebra tilt angles (UEVTA/LEVTA) (Fig. 2a). The parallel line AB is drawn through the vertex of Cobb angle O (Fig. 2b). The sideline of the Cobb angle that extended to line AB intersects at E, the parallel line CD is drawn through the vertex of Cobb angle O, and the sideline of the Cobb angle that extended to line CD intersects at F (Fig. 2c). It can be deduced in accordance with parallelogram law and supplementary angle law that:

$$ \angle \mathrm{Cobb}={180}^{{}^{\circ}}-\angle \mathrm{AEO}-\angle \mathrm{CFO}={180}^{{}^{\circ}}-\left({90}^{{}^{\circ}}-\alpha \right)-\left({90}^{{}^{\circ}}-\beta \right)=\alpha +\beta $$

The Cobb angle is the sum of upper and lower end vertebra tilt angles. The included angle of the upper vertebra endplate line with the horizontal line is measured on the imaging data (Fig. 2d), and the included angle of the lower vertebra endplate line with the horizontal line is measured on the imaging data (Fig. 2e). And then, the sum of two measured included angles is the Cobb angle (Fig. 2f).

### Time spent on measurement by end vertebra tilt angle

PACS spinal imaging pictures of this group of patients were exported and printed in A4 paper. After the upper and lower end vertebras and top vertebra were determined, three spine surgeons directly marked and measured the Cobb angle of the same scoliosis segment of the same patient using the classical method and tilt angle method, respectively, and then recorded the time spent on each measurement method using an electronic stopwatch and calculated the average value.

### Occupied pixel space marked by the end vertebra tilt angle measurement method

**Fig. 3:**
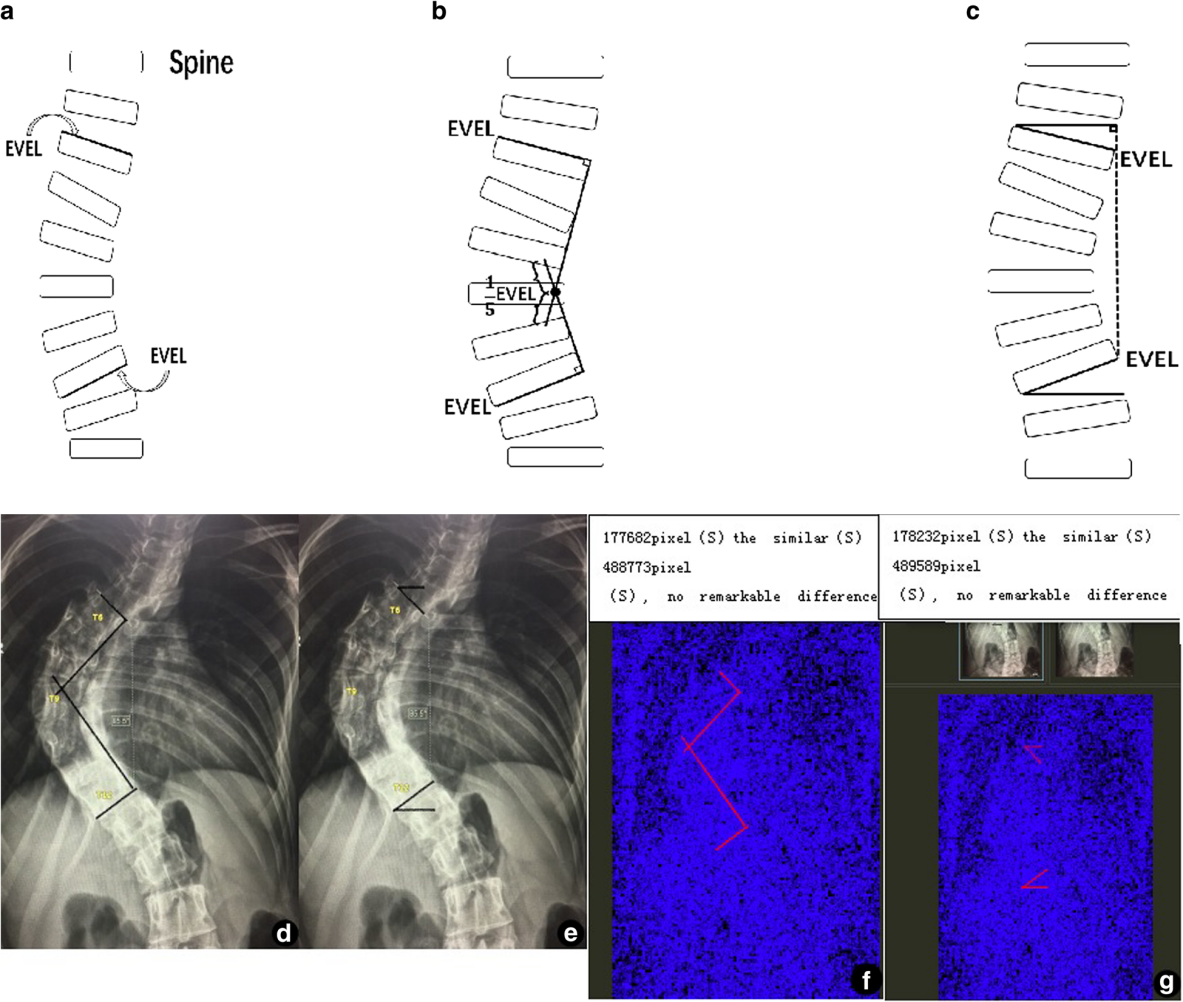
**a**, **b** Line marking of the classical method in a sketch map. **c** Line marking of the tilt angle method in a sketch map. **d** Line marking of the classical method in the imaging data. **e** Line marking of the tilt angle method in the imaging data. **f** Occupied pixel space marked by the classical method. **g** Occupied pixel space marked by the tilt angle method

The patient’s whole spine anteroposterior X-ray film was scanned and made into a figure with a resolution of 654 × 1024 pixels. The drawing tool in the same computer was used to simulate the line markings of the classical method and the tilt angle method to measure the Cobb angle, and the stroke parameters of all drawings were kept the same. The line markings for the same Cobb angle should meet the following requirements to facilitate quantitative comparison: (1) The EVEL line ends with both lateral margins of the vertebra endplate (Fig. 3a). (2) Two EVEL vertical lines of the classical method are intersected into an angle, and one fifth of the EVEL length is extended from the intersection point (Fig. 3b). (3) The starting point of the horizontal line of the tilt angle method is intersected with the starting point of the EVEL line, and the vertical line drawn at ending point could be exactly intersected with the ending point of the EVEL line (Fig. 3c). Figures marked with a line were imported, and the line markings of the two measurement methods were compared with Beyond Compare for Mac software. The occupied pixel space value and the difference value of the two groups were automatically calculated. Then, the visual interference degree of line markings of the two groups in the whole imaging data was compared (Fig. 3d–g).

### Statistical processing

Analysis was performed using SPSS 23.0 for Mac statistical software; data were expressed in mean value ± standard deviation. Complete random one-way ANOVA is used to test the results of the angle of the three methods whether there is any error. We select *t* test to compare whether there is a difference between the tilt angle method and the classical method in measuring time. To compare the measuring error between the tilt angle method and the classical method, whether there is statistical difference, we chose to use the Mann-Whitney *U* test. *p* < 0.05 means there is statistical significance for the difference.

## Results

The PACS measuring result regarded as the reference standard [2] was rounded to an integer to conform to an actual clinical application. To compare the results of the angle of the three methods (PACS method, end vertebra tilt angle method, classical method), whether there is any error, a complete random one-way ANOVA is used to test.

In the test, we also need pairwise comparison; we choose the Bonferroni method and homogeneity test of variance. The following results were obtained:

1. In the homogeneity test of variance, significant *p* > 0.1 was obtained, which indicated that the same variance could be used for single-factor ANOVA.
2. Adjust *α*′ = *α*/*m* = 2 × 0.05/3 × (3 − 1) = 0.167
3. Result of the PACS method: The range of Cobb angles for 50 cases was 25~125°, the median was 60°, the average value was 60.96 ± 21.08°, and 95% confidence interval was 54.9°, 66.7°. The tilt angle method result average was 61.34 ± 21.24°, and 95% confidence interval was 55.4°, 67.5°. The classical method result average was 61.90 ± 21.34° and 95% confidence interval was 55.9°, 67.9°. The results of single-factor analysis of variance showed *F* = 0.033, *p* = 0.967, indicating that there was no statistical difference between the three methods.
4. The Bonferroni method was used for pairwise comparison of the three methods (*p* > 0.05), and there was no statistical difference between the three methods, indicating that there was no difference in measuring the angle.

To compare whether there is a difference between the tilt angle method and the classical method in measuring time, we select *t* test and obtained the following results:

1. Correlation coefficient was 0.284 (*p* < 0.05).
2. We got *p* < 0.05 through *t* test. The average time spent for the tilt angle method was 12.98 ± 2.14 s with 95% confidence intervals of 12.37 s, 13.59 s. The average time spent for the classical method was 18.96 ± 2.65 s with 95% confidence intervals of 18.20s, 19.71 s, which indicated that the measuring time of the two methods was different.
3. We got the mean difference of the measuring angle time between the tilt angle method and the classical method which is − 5.98, indicating that the time required to measure the angle by the tilt angle method is faster.

**Fig. 4:**
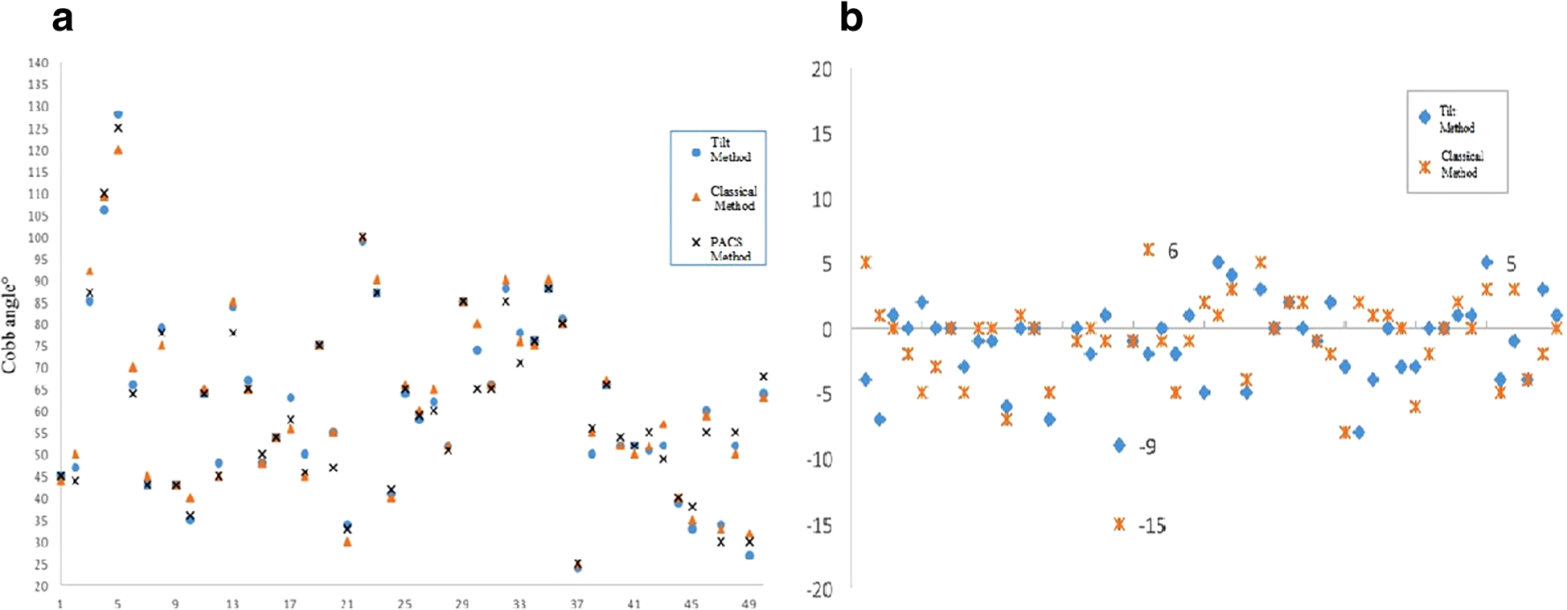
**a** Scatter diagram of Cobb angles measured by the three methods; X-axis represents the case numbers of the Cobb angle; Y-axis represents the Cobb angle. **b** Error scatter diagram of Cobb angles measured by the tilt angle method and the classical method; X-axis represents the case numbers of the Cobb angle; Y-axis represents the error range

To compare the measuring error between the tilt angle method and classical method, whether there is statistical difference, we chose to use the Mann-Whitney *U* test. The measuring error range was − 15° to approximately + 6° through the classical method with an average error of ± 3.67° and − 9° to approximately + 5° through the tilt angle method with an average error of ± 3.19°, which showed no statistical significance for the difference (*Z* = − 0.430; *p* = 0.667) (Fig. 4).

The software analysis of the pixel difference performed on picture results showed that the average pixel marked by the line drawing of the classical method was 3680 ± 533 pixels, accounting for 0.46~1.13% of total pixels, while the average pixel marked by the line drawing of the tilt angle method was 1539 ± 320 pixels, accounting for 0.12~0.32% of total pixels. Therefore, we can conclude that the pixel marked by the line drawing of the tilt angle method is less than that of the classical method, so the imaging data is less polluted.

## Discussion

Scoliosis is a three-dimensional deformity of the spine. No matter how complicated the scoliosis is, the measurement of the Cobb angle is based on the coronal or sagittal plane of imaging [3]. The Cobb angle is closely related to the spinous process angle of the coronal plane and rotation of the apical vertebra [4]. As for the bigger bending deformity of the spine coronal plane, the Cobb angle is the included angle of the upper end vertebra endplate line directly intersected with the lower end vertebra endplate line. For the smaller deformity of the spine, the intersected point of two endplate lines is outside of the X-ray film, so the vertical line of the upper end vertebra endplate line and that of the lower end vertebra endplate line shall be drawn to perform the measurement. In recent years, new measurement methods have been reported, such as the smartphone software [5, 6], PACS, and other computer software, and those methods are reliable and convenient and can replace the classical method to measure the Cobb angle [7–9]. In modern medical healthcare systems with digital radiographs and analyses, the idea of reducing drawing artifacts on an X-ray film is somewhat redundant. In developing countries, such as China, that still analyze radiographs on conventional X-ray films, steps for classical Cobb angle measurement are as follows: (1) Draw an endplate line between the two intersections of the end vertebra endplate and lateral margins on the film or a straight line drawn between the upper tangent of pedicles’ eyes in the same vertebra. (2) Measure the rectangle angle of the upper endplate line to draw the vertical line, and measure the rectangle angle of the lower endplate line to draw the vertical line. (3) Measure the included angle between two vertical lines (Cobb angle). The classical Cobb method needs a line drawn in a large range, and this will easily contaminate imaging data. In addition, limited by conditions of radiology departments of different hospitals and imaging film size, it is hard to include the whole spine segments into one film, and films shall be taken segment by segment. Thus, the measurement of the Cobb angle shall be performed by manually splicing the films into one figure, so there are inconveniences and figure angle deviations.

According to the geometry law, it can be deduced that the Cobb angle is the sum of upper and lower end vertebra tilt angles, so the Cobb angle can be calculated by measuring end vertebra tilt angles. No matter how serious the curvature of scoliosis is, and whether the scoliosis segments are in one imaging film, the Cobb angle can be calculated accurately and rapidly just by determining the two end vertebras and measuring the tilt angles. The measuring steps of the tilt angle method are as follows: (1) Draw the upper and lower end vertebra endplate connecting line on the film. (2) Measure the tilt angles of the upper and lower endplates. (3) Add the two measured results to get the Cobb angle. Obviously, the tilt method reduces one measurement step, so it can reduce the measuring time. In this study, the average time spent when using tilt angle to measure an angle is about 6 s less than that of the classical method. If you are skilled in the method, you can utilize the rectangular structure of a measuring ruler to fast determine the horizontal line and measure the end vertebra tilt angle in combination with the straight edge of the figure on the imaging film, which is more fast and convenient than the classical method in which the two vertical lines shall be additionally drawn for measurement. If the endplate connecting line develops clearly, the marker line of a measuring ruler can be directly utilized to perform overlap measurement, which can be free from the line drawing step.

When judging the interference and containment degree of line marking in imaging data, the figure treatment and analysis can be used to compare the difference of pixels marked by lines, which is more precise than visual observation and judgment [10]; the interference in pixels marked by the tilt angle is only 23.9~28.3% of that by the classical method, greatly lowering the contamination of line on imaging data.

**Fig. 5:**
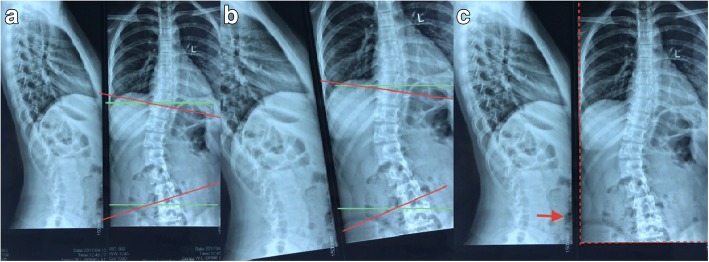
**a** Normally placed film. **b** Tilted placed film. **c** Red dotted line: the rectangular structure of the imaging data itself, and the ruler line (arrow) is vertical to the real horizontal plane

A previous study has suggested that the Cobb measurement method has several sources of errors [11]: non-standard position of patients or/and devices in imagological examination. To confirm the correct marker lines in the scoliosis segments which have anatomical variation of the vertebrae, different observers identify the different upper and lower end vertebrae. For those reasons, the measuring error range for the classical Cobb method was 6~9° [11, 12]. The tilt method is a methodological improvement based on the Cobb method, which has the same measuring errors as the former one. The efficacy and effectiveness of the tilt method were observed and compared by the same observers via using the same medical images. Therefore, the most common errors are intrinsic to the measurement method. The tilt method needs to draw two horizontal lines on the X-ray film. It is hard to make an accurate judgment about the reference points associated with the horizontal line of the actual torso. In addition, when the film is placed on the table or on the radiographic view box, the horizontal plane judgment will deviate from the real plane when the film is tilted, and the horizontal line is not the same as the horizontal of the actual torso. It is easy to make measuring error. But we found through computer simulation measurements that even though the film was tilted or the real horizontal plane was difficult to determine, there was no obvious measuring error. As shown, it is exactly the same case of scoliosis X-ray image data (Fig. 5a, b). We tilted the film to simulate the actual film placed on the table or on the film viewing illuminator, so the drawing line may deviate from the horizontal line. The green line is a horizontal line based on the entire imaging data, and it was given by the computer automatically. The red line was respectively at the upper/lower end vertebra endplate line. The angle between the red and green lines is an end vertebra tilt angle. The angle is completely consistent according to the geometry law and the actual observation (Fig. 5a, b).

Even though there is no statistical difference between the measurement error of the tilt method and the classical method, there are still some procedures to avoid the measurement error as far as possible. For instance, the shape of the imaging film is a rectangle, and the ruler line on the film is standard vertical or horizontal; it was given by the computer. We can use it as a reference point (Fig. 5c). On the other hand, we use the ruler as a measuring tool. The shape of the ruler is a rectangular structure, so we can make full use of the rectangular structure of the ruler and the rectangular outline of the imaging film as the reference point of the horizontal line. For example, the wide edge of the ruler overlaps with the edge of the film, and the line drawn on the long edge of the ruler must be the true horizontal line of the imaging data.

Cobb angle > 10° means that scoliosis exists, 10~25° means regular recheck shall be performed, and 25~45° means orthosis shall be needed. Cobb angle > 45° means surgical interference is needed. Cobb angle > 5° in two X-ray examinations indicates the scoliosis deformity progress [13]. Therefore, the measuring error for Cobb angle > 5° will possibly interfere with the diagnosis and treatment results. There is always a difference existing in the measurement of the Cobb angle of the same patient, and it is related to the patient position and photography angle. The manual line drawing and artificial observation are still the main reasons for measuring error. This study took the Cobb angle measured by PACS as the reference standard; the measuring error range for the classical method was − 15~ 6° with an average error of ± 3.67°, and the measuring error range for the tilt angle method was − 9~5° with the average error of ± 3.19°. The first reason for error is that the cases with complicated scoliosis we included were less; the second reason is that the imaging data included in the study were made into pictures of the same resolution and size in advance and printed and then UEV and LEV were determined uniformly for measurement and comparison [14], which could statistically reduce the measurer’s judgment bias and inter-group error.

## Conclusions

The scoliosis Cobb angle can be measured accurately and rapidly by the principle of the Cobb angle being equal to the sum of tilt angles of upper and lower end vertebra, wherein the film data of imaging will not be easily contaminated. Under special conditions, the average measuring error is ± 3°.

## Abbreviations

EVEL: End vertebra endplate lines
EVTA: End vertebra tilt angle method
LEV: Lower end vertebras
LEVEL: Lower end vertebra endplate lines
LEVTA: Lower end vertebra tilt angles
PACS: Picture archiving and communication systems
UEV: Upper end vertebras
UEVEL: Upper end vertebra endplate lines
UEVTA: Upper end vertebra tilt angles
